# Allergen-Specific IgE and IgG4 as Biomarkers for Immunologic Changes during Subcutaneous Allergen Immunotherapy

**DOI:** 10.3390/antib10040049

**Published:** 2021-12-07

**Authors:** Georgi Nikolov, Yana Todordova, Radoslava Emilova, Diana Hristova, Maria Nikolova, Bogdan Petrunov

**Affiliations:** Department of Immunology, National Center of Infectious and Parasitic Diseases, 1504 Sofia, Bulgaria; todorova_yana@ncipd.org (Y.T.); remilova@ncipd.org (R.E.); imlab@ncipd.org (D.H.); mstoimenova@ncipd.org (M.N.); petrunov@ncipd.org (B.P.)

**Keywords:** subcutaneous allergen-specific immunotherapy, biomarkers, specific IgE, specific IgG4, sIgE/sIgG4 ratio

## Abstract

(1) Background: Biomarkers of efficacy for subcutaneous immunotherapy (SCIT) on allergic rhinitis have not been evaluated in details. The present study aims to assess the relevance of measuring of sIgE, sIgG4 and IgE/IgG4 ratio during SCIT in patients with allergic rhinitis; (2) Methods: 20 patients, 13 men and 7 women aged 19 to 58 years, with clinically manifested seasonal and perennial allergic rhinitis were studied. At the initiation and in the end of the three-year course of SCIT serum allergen-specific IgE and IgG4 were measured with ImmunoCAP system. The sIgE/sIgG4 ratio was calculated as a biomarker for immunologic effectiveness; (3) Results: There was a significant increase of sIgG4 antibodies (*p* < 0.05), while at the end of SCIT for the sIgE levels no significant changes were seen (*p* > 0.05). Moreover, 90% of patients showed a decrease of the IgE/IgG4 ratio; (4) Conclusions: In most of treated patients with AR, SCIT with Bulgarian allergen products leads to clear immunological changes. After a 3-year of SCIT there is a significant increase in allergen specific IgG4 levels and both decrease of sIgE and IgE/IgG4 ratio. sIgE, sIgG4 and IgE/IgG4 ratio can be used as a substantial biomarker for predicting immunological effectiveness of SCIT.

## 1. Introduction

Allergen-specific immunotherapy (SIT) is the only treatment that can change the natural course of allergic diseases and is effective in allergic rhinitis (AR) or bronchial asthma [1]. The treatment improves symptom scores, decreases medication costs, and may prevent additional sensitization and progression to asthma [2].

Although the mechanisms of SIT are not fully understood, studies support the production of blocking antibodies, such as specific immunoglobulin G4 (sIgG4), as one of the key mechanisms of immunotherapy [3]. Upon initiation of immunotherapy, regulatory T cells cause B cells to produce sIgG4 instead of specific immunoglobulin E (sIgE). As a result, IgE-mediated allergic reactions are inhibited by competitive binding of IgG4 against the allergen. As a short-term effect, IgG4 starts to increase after one week and continues to increase for up to 1–3 years after treatment [4]. Therefore, as a long-term effect of immunotherapy IgE and IgE/IgG4 ratio decrease, whereas IgG4 increases [5].

The efficacy of immunotherapy is usually determined by clinical outcomes and laboratory findings: sIgG4 and sIgE levels as well as sIgE/sIgG4 ratio [6]. The abovementioned biomarkers, however, are not evaluated in details for SCIT of allergic rhinitis.

Having that in mind the present study aims to assess the relevance of measuring sIgE, sIgG4 and IgE/IgG4 ratio during SCIT in AR patients.

## 2. Materials and Methods

### 2.1. Subjects

The study included 20 patients, 13 men and 7 women aged 19 to 58 years, with clinically manifested IgE-mediated allergy (seasonal and perennial AR).

### 2.2. Allergy Diagnosis In Vivo

To confirm the specific sensitization of participants, a skin allergy test (prick test) was performed with a set of allergens, Bul Bio NCIPD Ltd., Sofia, Bulgaria, including: grass pollen mix, tree pollen mix, D. pteronyssinus, Alternaria alternata and cat allergen. Negative (Coca I solution) and positive (Histamine Dihydrochloride 1 mg/mL) controls were applied in parallel. The reactions obtained, were read at 20 min. according to size of wheal and flare. Size of wheal and flare with a mean diameter > 3 mm was considered positive.

### 2.3. Allergen-Specific Immunotherapy

All allergic patients, enrolled in the study, were referred for a course of subcutaneous specific hyposensitization with a relevant allergen, Bul Bio NCIPD Ltd. The selection of the allergenic preparation for SCIT is made according to the individual specific sensitization of each patient assessed by the case history and determination of specific IgE antibodies.

The allergens for specific immunotherapy are in liquid form whithout adjuvants. The basic therapy course injections were given strictly subcutaneously twice a week, according to a definite schedule. After completion of the basic course a maintaining course was administered with the highest allergen concentration reached as follows: 2–4 months × 0.4 mL once a week followed by 10–14 months × 0.4 mL once every 10 days. In case of good clinical effect, the interval between the injections was prolonged to 14–20 days.

The course of specific hyposensitization under monitoring lasted 3 years.

### 2.4. Allergen-Specific IgE and IgG4 Determination

At the initiation and in the end of the three-year course of subcutaneous immunotherapy serum samples from all participants in the study were collected. To avoid the effect of seasonal fluctuations in antibodies, especially of allergen-specific IgE to so-called seasonal allergens, the sera samples were collected at the same point time during the year outside the season of prevalence of the allergens in question.

Allergen-specific IgE to following allergens: g6 Phleum pratense; d1 Dermatophagoides pteronyssinus and m6 *Alternaria alternata* were measured in ImmunoCAP SystemTM (Phadia, Uppsala, Sweden) by FEIA-method according to manufacturer’s instructions.

The levels of sIgE were measured in kUA/L and were estimated by a 6 grade EAST scale. Positive reactivity was defined as sIgE level ≥0.35 kU/L (class 1 or above).

Allergen-specific IgG4 to the following allergens: Gg6 Phleum pratense; Gd1 Dermatophagoides pteronyssinus and Gm6 *Alternaria alternata* were measured in ImmunoCAP SystemTM (Phadia, Uppsala, Sweden) by FEIA-method according to manufacturer’s instructions.

The levels of sIgG4 were measured in the detection range 0.07–30 mg_A_/L, where A represents antigen-specific antibodies.

The ratio of sIgE/sIgG4 is calculated for each patient and then the sIgE/sIgG4 mean ratio is taken from individual values.

### 2.5. Statistical Analysis

All analyses were performed using Mann-Whitney test (GraphPad Prism 6.0, GraphPad Software, Inc., San Diego, CA, USA). *p* values of less than 0.05 considered as significant.

## 3. Results

### 3.1. Determination of Specific Sensitization of Patients with Respiratory Allergy

**Figure 1:**
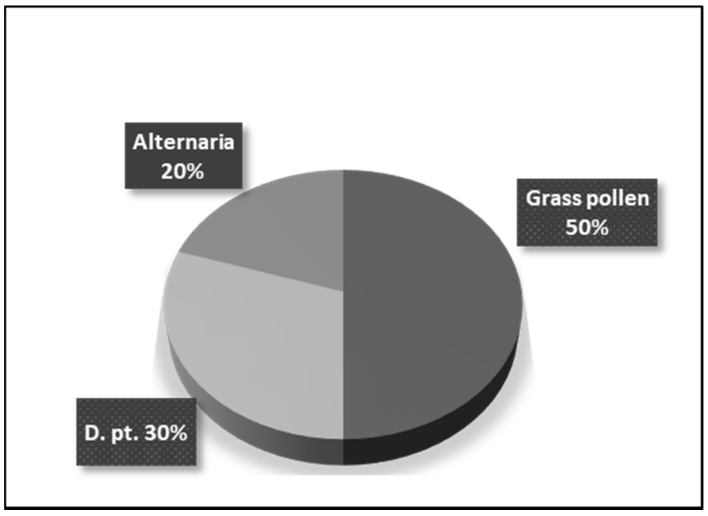
Specific sensitization of patients with allergic rhinitis.

Skin allergy testing revealed that 10 (50%) of the patients enrolled in the study were sensitized to grass pollen; 6 (30%)—to house dust mites and 4 (20%)—to Alternaria alternata (Figure 1). All patients were referred for a course of subcutaneous specific hyposensitization with the relevant allergen.

### 3.2. Changes of sIgE Levels after SCIT Using ImmunoCAP

We compared the immunologic parameters before and after 3 years SCIT. Before treatment, sIgE average levels to *Phleum pratense* (61.38 ± 23.36 kUA/L) were highest in patients with AR (Table 1).

At the end of SCIT no significant changes were seen in the levels for serum IgE specific for *Phleum pratense* or *Alternaria alternata*, while sIgE levels specific for house dust mite allergen *D. pteronyssinus* significantly decreased (*p* = 0.0260).

### 3.3. Changes of sIgG4 Levels after SCIT Using ImmunoCAP

A significant rise in allergen specific IgG4 in response to SCIT was observed in patients treated with grass pollen allergen (Table 2). The most significant responses were observed for *Phleum pratense* IgG4 from 0.09 ± 0.05 mg_A_/L before SCIT to 1.31 ± 0.63 mg_A_/L after SCIT, (*p* < 0.0001). *Alternaria alternata* IgG4 levels increased from 0.2 ± 0.14 mg_A_/L before SCIT to 0.30 ± 0.04 mg_A_/L after SCIT, (*p* > 0.05). In patients treated with house dust mite allergen the elevation of allergen-specific IgG4 to *D. pteronyssinus* was not significant (from 0.44 ± 0.43 mg_A_/L before SCIT to 0.71 ± 1.31 mg_A_/L after SCIT, *p* > 0.05).

### 3.4. sIgE/sIgG4 Ratio

**Figure 2:**
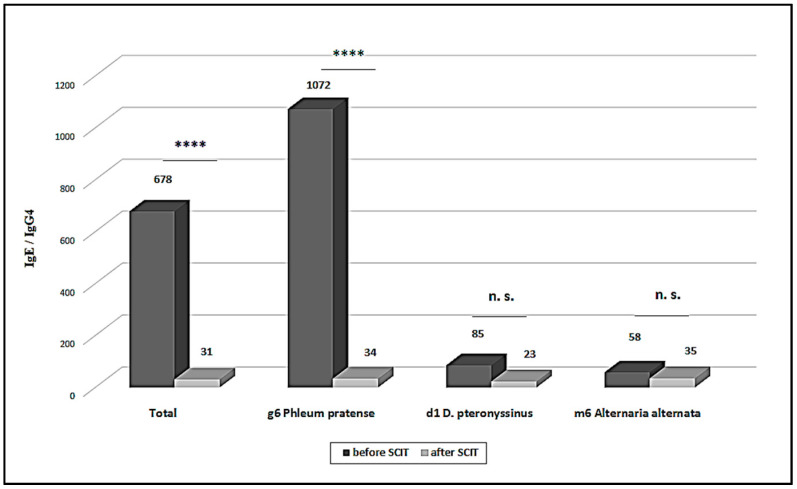
sIgE/sIgG4 ratio before and sfter SCIT. **** statistically significant difference (*p* < 0.0001); n.s. not significant.

After SCIT, the IgE/IgG4 ratio showed a significant decrease in patients treated with grass pollen allergen and allergen from *Alternaria alternata*. Again, the decline was most prominent in patients treated with grass pollen allergen (*p* < 0.0001) (Figure 2).

## 4. Discussion

Elevated serum specific IgE levels and symptoms upon exposure to the sensitizing allergen are currently the sole standard for allergy diagnosis and inclusion criteria for starting allergen immunotherapy, which is the only treatment option with disease-modifying properties and long-term clinical benefit after cessation [7]. Continuous subcutaneous immunotherapy over several years can result in a decrease of allergen-specific IgE concentrations, an event that might contribute to long-term tolerance. Furthermore, during the first months of SCIT, the serum levels of allergen-specific IgG4 have been shown to increase in a time- and dose-dependent manner [6,8].

Recent studies on immunotherapy with inhalant allergens suggest a protective role of IgG4 antibodies during treatment. Several studies have reported a 10- to 100-fold increases in serum concentrations of IgG, particularly IgG4, compared with baseline values during immunotherapy, although without consistent correlation with the clinical response to treatment [9], while others have observed a correlation between sIgG4 and clinical outcomes [10,11,12,13]. In fact, the induction of sIgG4 during AIT appears to be related to tolerance development.

The mechanism underlying the regulation of IgE and IgG4 production during SCIT is controversial. Th2 produced IL-4 induces both IgE and IgG4 switching in B cells [8], while IL-10 inhibits IgE production while up regulating the secretion of IgG4, suggesting different ways for control of IgE and IgG4 production [14].

sIgG4 is considered to compete with IgE for binding to allergen, thereby blocking allergen-IgE complex formation. This prevents cross-linking of high-affinity IgE receptors (FcεRI) on basophils and mast cells and inhibits histamine release. Competition between IgG/IgG4 and IgE can also block binding of allergen-IgE complexes to low-affinity receptors (FcgRIIb) on B cells, thereby inhibiting IgE-facilitated antigen presentation to T cells, a major driver of allergen-specific Th2 responses [15]. These distinct features suggest an anti-inflammatory role of sIgG4.

IgG4 antibodies are dynamic molecules that exchange Fab arms by swapping heavy–light chain pairs between IgG4 molecules with different specificities. This process results in the production of bispecific antibodies with a substantially decreased capacity for cross-linking, because they are functionally monomeric [15]. In addition, serum ‘blocking’ IgG4 antibodies have the capacity to suppress both allergen-triggered basophil histamine release and the binding of IgE–allergen complexes to B cells.

The problem of finding suitable surrogate paraclinical markers during SIT is currently very relevant. International guidelines for SIT highlight the need for quantitative and validated measurements for potential biomarkers. Biomarkers can be utilized to assist patient selection, identification of responders, and target intervention at those who will benefit and to exclude those who are less likely to respond to treatment as well as efficacy monitoring during intervention. Additionally, they can be of major importance for the development of novel vaccines and for the optimization of existing therapeutic regimes Biomarkers for SIT would thus facilitate the introduction of personalized medicine in allergy [16].

Although several biomarkers such as levels of sIgE and sIgG4, or sIgE/sIgG4 ratio have been included as secondary measurements in AIT studies, there are only very limited data on the relationship between biomarkers, immunologic and clinical response vs. nonresponse.

Regarding sIgE levels one study showed no significant difference [17], while several other studies reported significant decrease of sIgE after treatment [18,19].

Several studies demonstrated correlation between allergen-specific IgG4 and clinical outcomes [10,11,12,13]. Most studies, however, have shown an increase in IgG4 levels soon after the initiation of SIT followed by a decrease after cessation of treatment. Increases of IgG4 levels were associated with increase of interferon-gamma (IFN-γ), IL-10 and TGF-β [20,21].

The increase of IgG4 levels after SIT likely indicates an immunological response following allergen exposure during treatment and could potentially be used as a marker of therapy compliance reflecting the standardized preparation of the vaccine and effective administration. Low sIgG4 is a potential negative predictive marker. Failure in IgG4 induction may also be indicative of inadequate compliance.

The monitoring of sIgE/sIgG4 ratio can be used as biomarker for the efficacy of specific immunotherapy. In several SIT studies this ratio was decreased in correlation with decreased late-phase skin reactivity as a sign of immunological tolerance development via a modified Th2 response [8,13].

In the present study, we followed the changes of three potential biomarkers: sIgE; sIgG4 and sIgE/sIgG4 ratio during SCIT with Bulgarian allergens.

After 36 months of treatment there was a significant increase of IgG4 antibodies (*p* < 0.05), while no significant changes were observed for sIgE levels no significant changes were seen (*p* > 0.05). IgG4 induction appeared to precede IgE changes, in agreement with the possible role of the Treg/IL-10 response induction at the initial SIT phase. Moreover, 90% of patients showed a decrease of the IgE/IgG4 ratio.

Thus, the IgE/IgG4 ratio seems to be the immunological variable with the greatest size effect value, as compared to changes in sIgE or sIgG4 levels, alone.

## 5. Conclusions

This study shows that in most patients with AR, SCIT with Bulgarian allergen products leads to clear-cut immunological changes. After a 3-year of SCIT a significant increase in allergen specific IgG4 levels was observed together with decrease of both sIgE level and IgE/IgG4 ratio. sIgE, sIgG4 and IgE/IgG4 ratio can be used as indicative biomarkers predicting the immunological efficiency of SCIT.

## Figures and Tables

**Table 1:** Changes of sIgE levels after SCIT, mean (kUA/L) and standard deviation (SD).

Allergen	SCIT		*p*
	Before SCIT	After SCIT	
g6 Phleum pratense	61.38 ± 23.36	43.75 ± 33.71	0.2394
d1 D. pteronyssinus	21.18 ± 21.61	5.32 ± 4.21	0.0260 *
m6 Alternaria alternata	6.96 ± 5.06	10.07 ± 7.02	0.4571
TOTAL	38.44 ± 31.07	25.48 ± 30.00	0.1190

* statistically significant difference (*p* < 0.05).

**Table 2:** Changes of sIgG4 levels after SCIT, mean (kUA/L) and standard deviation (SD).

Allergen	SCIT		*p*
	Before SCIT	After SCIT	
g6 Phleum pratense	0.09 ± 0.05	1.31 ± 0.63	<0.0001 *
d1 D. pteronyssinus	0.44 ± 0.43	0.71 ± 1.31	0.4567
m6 Alternaria alternata	0.2 ± 0.14	0.30 ± 0.04	0.342
TOTAL	0.21 ± 0.28	0.93 ± 0.90	<0.0001 *

* statistically significant difference (*p* < 0.05).

## Data Availability

The datasets generated for this study are available on request to the corresponding author.
